# Transperineal template-guided saturation biopsy aimed at sampling one core for each milliliter of prostate volume: 103 cases requiring repeat prostate biopsy

**DOI:** 10.1186/s12894-017-0219-1

**Published:** 2017-04-05

**Authors:** Yasushi Nakai, Nobumichi Tanaka, Satoshi Anai, Makito Miyake, Shunta Hori, Yoshihiro Tatsumi, Yosuke Morizawa, Tomomi Fujii, Noboru Konishi, Kiyohide Fujimoto

**Affiliations:** 1 Department of Urology, Nara Medical University, 840 Shijo-cho, Kashihara-shi, Nara 634-8522 Japan; 2 Department of Pathology, Nara Medical University, 840 Shijo-cho, Kashihara-shi, Nara 634-8522 Japan

**Keywords:** Repeated prostate biopsy, Saturation biopsy, Transperineal template-guided biopsy

## Abstract

**Background:**

We evaluated the cancer detection rate of prostate cancer using transperineal template-guided saturation biopsy aimed at sampling one core for each milliliter of prostate volume for patients requiring repeated prostate biopsies.

**Methods:**

In total, 103 consecutive patients with repeated prostate biopsies were enrolled in this retrospective study. The number of biopsy cores was defined by prostate volume. In principle, one biopsy core covered 1 mL of prostate volume. We used a prostate brachytherapy template with a 5-mm grid and adopted a transperineal needle biopsy.

**Results:**

The median age, prostate-specific antigen level, and prostate volume were 69 (range, 37–83) years, 9.2 (range, 1.9–107) ng/mL, and 34.7 (range, 18–76.7) mL, respectively. The median number of biopsy cores was 37 (range, 18–75 cores). Fifty-three patients (51.5%) were diagnosed with prostate cancer. The Gleason score was 6, 7, and 8–10 in 24.5, 64.2 and 11.3% patients, respectively. Forty-two patients (79.2%) were diagnosed with clinically significant PCa. Acute urinary retention was detected in 2 patients (1.9%).

**Conclusions:**

Transperineal template-guided saturation biopsy with one core per milliliter of prostate volume helped achieve a high cancer detection rate and high significant cancer detection rate with acceptable biopsy-associated adverse events.

## Background

Prostate-specific antigen (PSA) testing has been widely used for prostate cancer (PCa) screening, and transrectal ultrasound (TRUS)-guided biopsies have been widely performed [1, 2]. However, even with contemporary use of laterally directed extended TRUS-guided biopsies, the false-negative rate remains high [3]. Patients with negative diagnosis by TRUS-guided biopsy may need repeat biopsy if the following findings are present: increased PSA levels; abnormal findings on digital rectal examination (DRE), TRUS, and MRI; and previous biopsy showing high-grade prostatic intraepithelial neoplasia (HGPIN) and/or atypical small acinar proliferation (ASAP). Cancer detection rates with TRUS-guided repeat biopsy have been reported to range from 10 to 21% [4–6]. These results indicate that patients continue to be under persistent clinical suspicion of PCa despite several repeated biopsies. To resolve this problem, several investigators have reported the use of saturation biopsy [7–12].

Prostate saturation biopsy was initially introduced by Borborogle et al. [7]; it consisted at least 20 biopsy cores. Saturation biopsy is performed via transrectal or transperineal routes, with similarly high detection rates [8–13]. Recently, the transperineal approach has been preferred because of sampling accuracy, particularly for the anterior prostate region [8–10]. Although the technique of transperineal saturation biopsy has varied among reports, the cancer detection rate has been reported to be between 40 and 60% in repeated biopsies [8–10, 14, 15]. Previous studies on saturation repeat biopsy showed higher detection rates than those on TRUS-guided repeat biopsy. However, the optimal number of biopsy cores remains in dispute. Buskirk et al. [16] showed that the number of needle incursions was the only prognostic factor of acute urinary retention (AUR). The incidence of AUR has been reported to be 10–39% [10, 17–19]. To prevent AUR, Ekwueme et al. [9] demonstrated the efficacy of transperineal saturation biopsy to avoid a needle incursion in the periurethral region, and AUR rate was found to be only 5.2%. Furthermore, McNeal and Chen et al. reported that PCa is rarely detected in the periurethral region [20, 21].

Under these circumstances, we conducted a study to evaluate the efficacy and safety of transperineal template-guided saturation biopsy (TTSB) aimed at sampling one core for each milliliter of prostate volume for patients who had undergone at least one negative TRUS-guided biopsy and requiring repeated prostate biopsy for increased PSA levels, abnormal findings on DRE, TRUS, and MRI, previous biopsy showing HGPIN, or ASAP.

## Methods

### Patient selection

From January 2008 to July 2014, we offered transperineal TTSB for patients considered to need prostate repeat biopsy when the following clinical factors were present: increased PSA levels; abnormal findings on DRE, TRUS, or MRI; and a previous biopsy showing HGPIN and/or ASAP. The number of previous TRUS-guided biopsies from TTSB showed 1–5 negative biopsies (median: 1). A total of 103 consecutive patients who received TTSB repeated prostate biopsy were enrolled in this study. Then we retrospectively analyzed the data. The institutional review board of the Nara Medical University approved this study.

### Procedure of TTSB

**Fig. 1:**
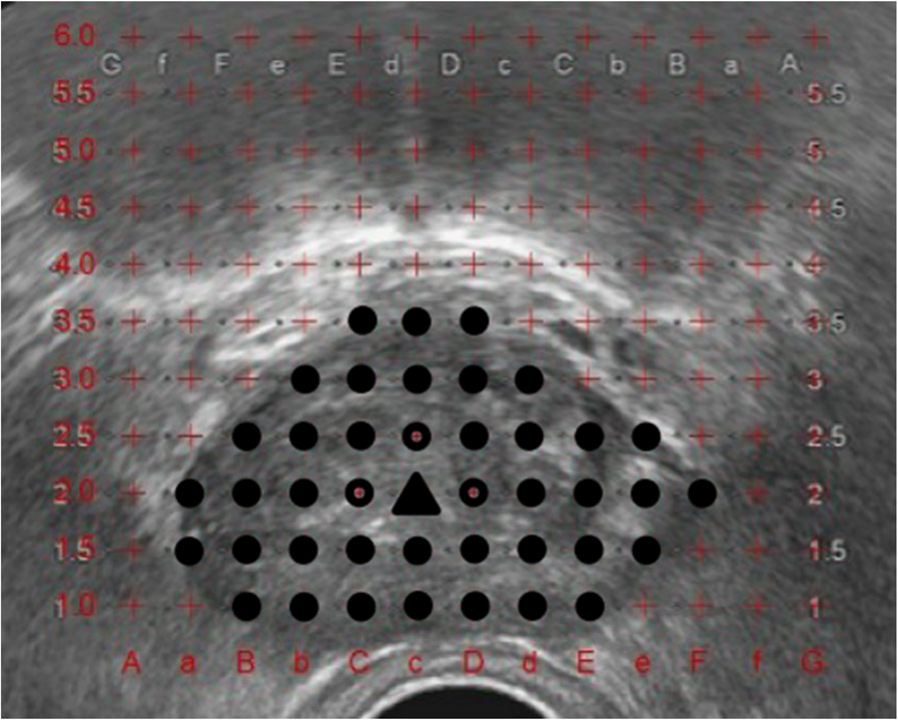
Ultrasound image of the prostate showing the sites for sampling (*black dots*). *Triangle* shows urethra. *Circle* shows sites where sampling was avoided

All procedures were performed in the operating room. TTSB was performed in the dorsal lithotomy position under either general or spinal anesthesia; a 14-French urethral catheter was inserted before the procedure. Every patient received premedication with a single dose of 1 g cefazolin by intravenous infusion for preventing infection caused by TTSB. DRE was performed, and a transrectal probe (Toshiba Medical, Tochigi, Japan) attached to a brachytherapy stepping unit (AccuSeed, Bedfordshire, UK) was then inserted into the rectum. The low-echoic area was estimated and prostate volume was calculated using the following formula: length × width × height × 0.5236 [22]. The number of biopsy cores was estimated using the widest transverse section (Fig. 1). The interval between biopsy cores in a row was uniformly 5 mm in rows from right to left in the longitudinal view, except for the area nearest to and around the urethra. At a point where sufficient sample was not taken from the apex of the bladder, an additional core was considered to take the sample from a point near the bladder. The number of additional cores taken was determined by the calculated prostate volume, and the number of biopsy cores was determined based on prostate volume. To achieve a “saturation biopsy,” one biopsy core per milliliter of prostate volume was required. The biopsy procedure was performed using an 18-gauge, 25-cm-long biopsy gun (Bard, Covington, GA, USA).

After TTSB, patients were monitored in the hospital until the next day noon. The next morning, the urethral catheter was removed. Patients were discharged once they could void successfully.

PSA density (PSAD) was calculated by dividing PSA by prostate volume. PSA velocity (PSAV) was defined as absolute increase in PSA level per year following the initial biopsy until the second biopsy. For patients with multiple PSA measurements prior to a saturation biopsy, we used the most recent value before the saturation biopsy date. Insignificant cancer was defined as a clinically insignificant Gleason score of 3, maximum tumor length of <4.5 mm, and total tumor length of <5.5 mm based on the report by Epstein [23].

### Statistical analysis

Statistical analysis was performed with SPSS for Windows (version 20.0; IBM, Armonk, NY, USA). Pearson’s correlation was calculated between pairs of variables. A Mann-Whitney U test was used for continuous variables and a chi-square test for categorical variables. Binary logistic regression analysis was used to estimate the independent parameter of positive TTSB. The cutoff value was determined as the point closest to the upper left-hand corner in the receiver operating characteristic curve. Univariate analysis was first applied to isolate variables with a significant value of *P* < 0.05. Variables predictive of PCa by univariate analysis were included in multivariate analysis. A *P* value <0.05 was considered statistically significant.

## Results

The median age, median PSA level, and median prostate volume were 69 (range, 37–83) years, 9.2 (range, 1.9–107) ng/mL, and 34.7 (range, 18–76.7) mL, respectively. The median number of previous TRUS biopsies sets was 1 (range, 1–5) and that of cores obtained by previous TRUS-guided biopsies was 12 (range, 8–13). TTSB resulted in a median of 37 cores (range, 18–75). Of the 103 patients, PCa was detected in 53 (51.5%). In 57 patients with gray-zone PSA (4–10 ng/ml), PCa was detected in 25 (43.9%). Patient age with positive biopsy was significantly higher than that with negative biopsy (*P* < 0.01), and PSAD of positive biopsy patients was significantly higher than that of negative biopsy patients (*P* = 0.02). Free PSA ratio of positive was significantly lower than that of negative biopsy patients (*P* = 0.04), abnormal findings on DRE were found more in positive biopsy patients than in negative (*P* = 0.03), and prostate volume of positive biopsy patients was significantly smaller than that of negative biopsy patients (*P* < 0.01). MRI was performed for 14 patients between the previous TRUS-guided biopsy and TTSB, and 9 patients underwent saturation biopsy because of abnormal findings on MRI. Six of nine patients (66.7%) were diagnosed with PCa on TTSB (Table 1)

**Table 1:** The clinical and pathological features of patients

Variables Median (range) or n (%)	Total *n* = 103	Prostate cancer *n* = 53	No prostate cancer *n* = 50	*P*
Age, years	69 (37–83)	71 (48–79)	67 (37–83)	<0.01^†^
No. of TRUS biopsies	1 (1–5)	1 (1–5)	1 (1–3)	0.48^†^
No. of cores obtained pre-TRUSBs	12 (8–13)	12 (8–13)	12 (8–12)	0.89^†^
PSA, ng/mL	9.2 (1.9–107)	10.7 (1.9–42.1)	8.8 (4.5–107)	0.33^†^
Free-PSA ratio, %	15.3 (1.3–67.1)	12.9 (4.3–25.7)	16.9 (1.3–67.1)	0.04^†^
PSA density, ng/mL/mL	0.26 (0.06–1.9)	0.33 (0.06–1.3)	0.23 (0.07–1.9)	0.02^†^
PSA velocity, ng/mL/year	1.12 (−29–129)	1.39 (−29–26)	0.75 (−8.8–129)	0.66
DRE, abnormal findings	17 (16.5)	13 (24.5)	4 (8.0)	0.03^‡^
TRUS, abnormal findings	21 (20.3)	14 (26.4)	7 (14.0)	0.13^‡^
MRI, abnormal findings (*n* = 14)	9/14 (64.2)	6/9 (66.7)	3/5 (60.0)	0.93^‡^
Prostate volume, mL	34.7 (18–76.7)	30.8 (18–65.1)	39.4 (20.1–76.7)	<0.01^†^
HGPIN on previous biopsy	13 (12.6)	7 (13.2)	6 (12.0)	0.88^‡^
ASAP on previous biopsy	10 (9.7)	8 (15.1)	2 (4.0)	0.06^‡^
No of cores obtained by TTSB	37 (18–75)	32 (22–66)	41.5 (18–75)	<0.01^†^
Core/volume, cores/mL	1.05 (0.80–1.77)	1.05 (0.80–1.31)	1.05 (0.80–1.77)	0.07^†^

^†^Mann-Whitney U test, ^‡^Chi-squared test

*TRUS* transrectal ultrasound, *PSA* prostate specific antigen, *DRE* digital rectal examination, *HGPIN* high grade prostatic intraepithelial neoplasia, *ASAP* atypical small acinar proliferation, *TTSB* transperineal template-guided sasturation biopsy, *Core/volume* number of cores per unit volume of prostate

Table 2 summarizes the number of positive cores and Gleason biopsy score. The distribution of positive cores was the most heavily weighted in those patients with 2–5 positive cores (50.1%). Gleason score distribution ranged from 6 to 9 with the vast majority 7 (64.2%). Eleven patients (20.8%) were diagnosed with clinically insignificant PCa.

**Table 2:** The number of positive cores and cancer grade

	Count n (%) *n* = 53
No. of positive cores	
1	11 (20.8)
2–5	27 (50.1)
6–9	9 (17.0)
≥ 10	4 (7.5)
Gleason score	
6	13 (24.5)
7	34 (64.2)
8	4 (7.5)
9	2 (3.8)

In the cores taken from the posterior and anterior regions of the prostate, PCa was detected in 33/53 (62.2%) and 44/53 (83%) cases, respectively. The incidence of PCa in the anterior region of the prostate was significantly higher than that in the posterior (*P* = 0.01). GS ≥ 7 PCa was found in 24/53 (45.2%) and 31/53 (58.5%) cases in the cores taken from the posterior and anterior regions of the prostate, respectively. There was no significant difference (*P* = 0.14 ) in the incidence of GS ≥ 7 PCa between the posterior and anterior regions.

Age, free PSA ratio, PSA density, abnormal findings on DRE, and prostate volume were the significant factors in univariate analysis. In multivariate analysis, prostate volume was a negative predictor of positive PCa by TTSB (≤34.1 vs. > 34.1 mL, *P* = 0.03) and age was a positive predictor of positive PCa by TTSB (≤70.3 vs. > 70.3 years, *P* < 0.01) (Table 3).

**Table 3:** Univariate and multivariate analysis of factors that predict positive for prostate cancer

Variables	Univariate analysis		Multivariate analysis	
Categories	Odds ratio (95% CI)	*P*	Odds ratio (95% CI)	*P*
Age				
≤ 70.3	(Ref)		(Ref)	
> 70.3	3.80 (1.68–8.61)	<0.01	4.96 (1.67–14.7)	<0.01
No. of TURSBs				
≤ 1	(Ref)			
> 1	0.70 (0.31–1.60)	0.78		
No. cores obtained by pre-TURSBs				
≤ 10	(Ref)			
> 10	1.01 (0.87–1.16)	0.91		
PSA				
≤ 9.3	(Ref)			
> 9.3	1.85 (0.83–4.07)	0.12		
Free PSA ratio				
≤ 13.6	(Ref)		(Ref)	
> 13.6	0.33 (0.14–0.78)	0.01	0.45 (0.13–1.60)	0.22
PSA density				
≤ 0.27	(Ref)		(Ref)	
> 0.27	2.69 (1.20–6.01)	0.02	2.03 (0.468–6.01)	0.20
PSA velocity				
≤ 1.2	(Ref)			
> 1.2	1.91 (0.86–4.25)	0.11		
DRE				
Benign	(Ref)		(Ref)	
Suspicious	3.73 (1.12–12.3)	0.03	2.87 (0.72–11.5)	0.13
TRUS				
Benign	(Ref)			
Suspicious	2.20 (0.81–6.03)	0.12		
Prostate volume				
≤ 34.1	(Ref)		(Ref)	
> 34.1	0.24 (0.11–0.55)	<0.01	0.28 (0.09–0.90)	0.03
HGPIN on previous biopsy				
Absent	(Ref)			
Present	1.11 (0.35–3.58)	0.85		
ASAP on previous biopsy				
Absent	(Ref)			
Present	4.23 (0.86–21.2)	0.08		
Core/volume				
≤ 1.05	(Ref)			
> 1.05	3.28 (0.41–24.5)	0.26		

*TRUSB* transrectal ultrasound -guided biopsy, *PSA* prostate specific antigen, *DRE* digital rectal examination, *TRUS* transrectal ultrasound, *HGPIN* high grade prostatic intraepithelial neoplasia, *ASAP* atypical small acinar proliferation, *TTSB* transperineal template-guided sasturation biopsy

There was no case of urosepsis and urinary tract infection. Only 2 of 103 (1.9%) patients needed catheterization after removal of the catheter. From one patient the urethral catheter could be removed after 6 days and from another after 10 days. Prostate volumes of the two patients were 73.1 and 46.0 mL. One patient had habitually used an alpha-1 adrenogenic receptor antagonist for benign prostatic hyperplasia.

## Discussion

The overall cancer detection rate of the present study was 51.5%; this was comparable to that reported in the previous studies (26–68%) [8–10, 15, 18, 22, 24–29]. Studies including more than 100 cases have reported detection rates of 35.6–54.8% [9, 10, 18, 22, 28, 29]. Although the technique of TTSB and the number of biopsy cores varied among these studies, the cancer detection rate in repeated biopsy patients was almost similar.

Significant cancer by saturation biopsy was defined by Epstein et al. as follows: (a) Gleason score less than 7 and the number of positive cores as three or fewer or (b) Gleason score less than 7 and the maximal millimeters of cancer in one core less than 4.5 mm, with the total millimeters of cancer for all cores not exceeding 5.5 mm [23]. Previous studies have found that incidence rates of clinically significant cancer were 85.1 and 86.7% based on the Epstein’s criteria [9, 24]. The present result (79.8%) was lower than those of these previous studies. Although increasing the number of cores may contribute to the high detection rate of insignificant cancer, the rate of detection of significant cancer in the present study was still high. Based on these results, TTSB should be considered for patients with persistent clinical suspicion of PCa without a negative previous biopsy.

In the present study, prostate volume showed a strong correlation with number of biopsy cores, and the mean number of biopsy cores per unit prostate volume was 1.06. This number is the highest in reports about TTSB [8–10, 13, 15, 18, 24–27]. However, the rate of detection of PCa was almost the same as that in other reports. This finding indicates that it is not necessary to sample as many cores as in our methods for detecting PCa. Subsequently, the number of cores may be reduced than that in our method. However, the optimal number of cores for saturation biopsy should be evaluated in the future.

Prostate volume has been considered a negative predictor of positive PCa by transperineal prostate biopsy in other reports [10, 28]. Merrick et al. used a 24-region technique with a median (mean, range) of 50.0 (51.1, 24–66) biopsy cores for patients whose mean prostate volume was 78.6 ml [10]. Symons et al. used a 14-region technique with a median (mean, range) of 15 (19.2, 4–47) biopsy cores for patients whose mean prostate volume was 45.8 ml [28]. In the present study, larger prostate volume predicted negative for PCa [odds ratio (OR), 0.29; 95%CI, 0.09–0.90] with a median (mean, range) of 37 (40.2, 18–75) biopsy cores for patients whose mean prostate volume was 38.5 ml. Even with this high number of biopsy cores per volume, large prostate volume was a negative predictor. This finding means that patients who have large prostates do not tend to have PCa. Based on these results and the detection rate with the high number of cores per volume in the present study, we can omit repeat biopsy for patients requiring repeated biopsy with large prostate. However, the positive predictive factors in the population of patients with large prostate should be determined in the future.

Multivariate analysis showed that age was a predictor of positive PCa. The median age of the patients (70 years) was higher than that in other reports [8–10]. It is believed [29] that the detection rate of PCa is higher in elderly than in younger men.

The reported incidence of AUR from TTSB ranged from 10 to 39% [10, 16–19] and obtaining more biopsy cores appears to have led to a high risk of AUR. In a previous study, Buskirk et al. [15] showed the relationship between needle trauma and AUR after TTSB. To prevent AUR, Ekwueme et al. [9] showed the safety of TTSB at 10-mm intervals by avoiding the periurethral region and reported the incidence of AUR as 5.9%. We obtained a low AUR rate of 1.9% by performing TTSB as described by Ekwueme et al., although we obtained more biopsy cores. Possible reasons for low rate of AUR are as follows: (a), catheterization overnight for all patients (b), smaller prostate volume in the patient population than other reports of saturation biopsy, and (c) avoiding insertion of needle to the periurethral region. In other studies, all patients were not catheterized overnight. [10, 11, 17–19] Prostate volume is a predictive factor for AUR [9]. AUR rate may rise in patient groups that include patients with larger prostates. However, in the present study, the mean prostate volume was smaller than that in other reports.

The present study had several limitations. First, it was retrospective study. Second, the median prostate volume in this study was smaller than that in other reports from Western countries. Indeed, the prostate volume of Japanese patients is smaller than that in Western populations [30]. Third, a control group of standard re-biopsy was lacking. The final limitation was the small cohort size.

## Conclusions

We demonstrated the feasibility of TTSB aimed at sampling one core for each milliliter of prostate volume for patients with persistent clinical suspicion of PCa who had undergone at least one negative TRUS biopsy. A relatively high cancer detection rate (51.5%) and significant cancer detection rate (79.8%) could be achieved with a low AUR rate (1.9%).

## Abbreviations

ASAP: Atypical small acinar proliferation
AUR: Acute urinary retention
DRE: Digital rectal examination
PCa: Prostate cancer
PSA: Prostate-specific antigen
PSAD: Prostate-specific antigen density
PSAV: Prostate-specific antigen velocity
TRUS: Transrectal ultrasound
TTSB: Transperineal template-guided saturation biopsy
